# Short-term water stress responses of grafted pepper plants are associated with changes in the hormonal balance

**DOI:** 10.3389/fpls.2023.1170021

**Published:** 2023-04-25

**Authors:** Yaiza Gara Padilla, Ramón Gisbert-Mullor, Salvador López-Galarza, Alfonso Albacete, Purificación A. Martínez-Melgarejo, Ángeles Calatayud

**Affiliations:** ^1^ Departamento de Horticultura, Instituto Valenciano de Investigaciones Agrarias, Moncada, Valencia, Spain; ^2^ Departamento de Producción Vegetal, Centro Valenciano de Estudios sobre el Riego (CVER), Universitat Politècnica de València, Valencia, Spain; ^3^ Department of Plant Nutrition, Centro de Edafología y Biología Aplicada del Segura (CEBAS-CSIC), Murcia, Spain; ^4^ Institute for Agro-Environmental Research and Development of Murcia (IMIDA), Department of Plant Production and Agrotechnology, Murcia, Spain

**Keywords:** *Capsicum annuum*, drought stress, leaves, phytohormones, root, water use efficiency

## Abstract

Phytohormones play an important role in regulating the plant behavior to drought. In previous studies, NIBER^®^ pepper rootstock showed tolerance to drought in terms of production and fruit quality compared to ungrafted plants. In this study, our hypothesis was that short-term exposure to water stress in young, grafted pepper plants would shed light on tolerance to drought in terms of modulation of the hormonal balance. To validate this hypothesis, fresh weight, water use efficiency (WUE) and the main hormone classes were analyzed in self-grafted pepper plants (variety onto variety, V/V) and variety grafted onto NIBER^®^ (V/N) at 4, 24, and 48h after severe water stress was induced by PEG addition. After 48h, WUE in V/N was higher than in V/V, due to major stomata closure to maintain water retention in the leaves. This can be explained by the higher abscisic acid (ABA) levels observed in the leaves of V/N plants. Despite the interaction between ABA and the ethylene precursor, 1-aminocyclopropane-1-carboxylic acid (ACC), in relation to stomata closure is controversial, we observed an important increase of ACC at the end of the experiment in V/N plants coinciding with an important rise of the WUE and ABA. The maximum concentration of jasmonic acid and salicylic acid after 48h was found in the leaves of V/N, associated with their role in abiotic stress signaling and tolerance. Respect to auxins and cytokinins, the highest concentrations were linked to water stress and NIBER^®^, but this effect did not occur for gibberellins. These results show that hormone balance was affected by water stress and rootstock genotype, where NIBER^®^ rootstock displayed a better ability to overcome short-term water stress.

## 1 Introduction

Drought stress is one of the most important environmental factors negatively affecting agriculture production and it has been aggravated in the last decade by climatic change worldwide (Gray and Brady, 2016). Most crops are highly vulnerable to drought stress, resulting in growth and production impairment with relevant economic consequences (Vicente-Serrano, 2007).

Plants have developed several adaptive strategies to mitigate the negative effects of water scarcity, evolving morpho-physiological, phenological, biochemical, and genetic mechanisms (Basu et al., 2016; Ullah et al., 2018). Plant roots are the first organs sensing soil water deficit and this perception induces a complex signaling network from root to shoot (and shoot to root), in which hormones, reactive oxygen species (ROS), sugars, other metabolites, and small nucleotides are mainly involved (Albacete et al., 2014). Among them, phytohormones are the key mediators of plant responses to drought stress, they are involved in the tolerance strategies (Pérez-Alfocea et al., 2011; Ullah et al., 2018) by producing chemical messengers which activate various physiological processes to overcome drought stress (Fahad et al., 2015).

Drought provokes osmotic stress that induces abscisic acid (ABA) synthesis, which is implicated in the synthesis of compatible osmolytes, the regulation of drought-responsive genes expression, and the regulation of stomatal closure. Generally, ABA synthesis occurs in the roots from where it is translocated to the leaves *via* the xylem sap, inducing stomatal closure to decrease water loss. However, several experiments have demonstrated that ABA biosynthesis also takes place in leaves (Holbrook et al., 2002; Manzi et al., 2017; López-Serrano et al., 2020), but also stomatal closure can occur without the assistance of ABA root synthesis (Holbrook et al., 2002).

Ethylene or its direct precursor 1-aminocyclopropane-1-carboxyl acid (ACC) is highly mobile within the cell and can be translocated basipetally *via* the phloem or acropetally through the xylem (Druege, 2006). Both have been considered important regulators of water stress responses by inducing leaf senescence, epinasty, organs abscission, and leaf growth inhibition (Acosta-Motos et al., 2020; Fatma et al., 2022).

Other hormones, such as auxins (IAA), cytokinins (CKs), and gibberellins (GAs) are directly involved in the control of plant growth and their concentrations can be environmentally modulated (Werner et al., 2001; Sachs, 2005), playing critical roles during water stress. However, they can have an opposite effect since high auxin levels have been associated with drought tolerance, while GA accumulation decreased tolerance (Ullah et al., 2018). CKs have shown a dual role under water stress since positive but also negative effects on drought tolerance have been reported (Zwack and Rashotte, 2015; Li et al., 2016). A decrease in CK transport from the root to the shoot could inhibit leaf growth while a low CK content would promote root growth and modify the root/shoot ratio (Rahayu, 2005).

Furthermore, jasmonic acid (JA) and salicylic acid (SA) are hormones classically involved in biotic stress tolerance signaling (Li et al., 2003), and it is only recently that their importance in abiotic stress responses has been revealed (Muñoz-Espinoza et al., 2015). Water deficit increased JA levels in several species (Brossa et al., 2011; Chen et al., 2016; de Ollas et al., 2018). Moreover, JA and SA regulate stomatal conductance, increase root hydraulic conductivity, enhance the scavenging of ROS by antioxidant activity stimulation, and promote root development, thus contributing to drought tolerance (Munné-Bosch and Peñuelas, 2003; Saruhan et al., 2012; Aslam et al., 2021). Their function is directly related to their relative and absolute concentrations, when SA and JA were equally applied externally at low concentrations they acted synergistically, whereas applying high concentrations of one hormone antagonized the other one (Mur et al., 2006).

It is important to note that the role of each phytohormone has been frequently described considering individual signaling pathways and not the hormonal interaction network, the spatial organ distribution, the long-distance hormonal signaling, and the type of crosstalk between hormones (positive or negative), which can be dependent of the magnitude (time and intensity) of the water stress. Indeed, different studies have demonstrated the hormonal interactions taking place under drought stress, highlighting the complexity of hormonal signaling cascades (Davies et al., 2005; Muñoz-Espinoza et al., 2015; de Ollas et al., 2018; Ullah et al., 2018; Devireddy et al., 2021; Huntenburg et al., 2022).

An important approach for discovering how long-distance hormonal communication and how roots can alter the shoot perception (or vice versa) under stress is the use of grafted plants. Vegetable grafting has become an effective strategy to increase tolerance under water stress (Rouphael et al., 2008; Penella et al., 2014; Sánchez-Rodríguez et al., 2016; López-Serrano et al., 2019; Gisbert-Mullor et al., 2020; Padilla et al., 2021) by the use of tolerant rootstocks that improve the physiological performance of plants under drought conditions. Some studies have demonstrated that the efficiency of tolerant rootstocks in overcoming water stress is related to their higher capacity to absorb water and nutrients, maintain root growth, achieve an active osmotic adjustment, and activate the antioxidant defense systems (Rouphael et al., 2008; Yao et al., 2016; Zhang et al., 2019a). This higher root efficiency under water stress contributes to maintain the metabolic processes taking place in the scion, sustaining plant growth and productivity. In addition, hormonal communication plays an important role in achieving water stress adaptation of grafted plants. Different combinations of rootstocks and scions have different modes of phytohormone synthesis transport (Lacombe and Achard, 2016; Lu et al., 2020) and affect plant adaptability to stress. ABA is the main hormone studied in grafted plants under water stress, because of its function in controlling stomata closure. Most studies have been done in tomato (Holbrook et al., 2002; Dodd et al., 2009; Ghanem et al., 2011; Cantero-Navarro et al., 2016; Gaion et al., 2018; Zhang et al., 2019b) and cucumber (Liu et al., 2016). However, there are no reports on hormonal balance regulation in grafted pepper plants exposed to water stress, being sweet pepper an important vegetable crop, thoroughly cultivated in the Mediterranean area, where water shortage is a major problem limiting productivity (Penella et al., 2014). Even more, the availability of rootstocks tolerant to water stress is lacking in pepper plants (Lee et al., 2010; Penella et al., 2014; Kyriacou et al., 2017). Nevertheless, to fill this gap we have obtained by a classic breeding program a water stress tolerant rootstock, NIBER^®^, an F1 hybrid that has been tested under field conditions achieving greater yields than the ungrafted variety (Gisbert-Mullor et al., 2020). Mechanisms by which NIBER^®^ rootstock modulates plant performance under water stress, particularly hormonal balance, neither have not been fully unraveled.

Therefore, the present work aimed to determine whether the water stress tolerance observed in pepper plants grafted onto NIBER® in terms of productivity is associated with changes in the hormonal balance in early stage of grafted plant development and identify the hormones role responsible for the drought tolerance in rootstock and scion. Understanding the interactive hormonal mechanism can be effective for the development to tolerant rootstocks.

To fulfill this, we compared the hormonal profiles (ACC, CKs, GAs, ABA, IAA, JA, and SA) in roots and leaves of two pepper graft combinations (variety grafted onto NIBER^®^ and self-grafted variety) under optimal and short-term water stress conditions.

## 2 Materials and methods

### 2.1 Plant material

Based on our previous studies (Gisbert-Mullor et al., 2020), a hybrid pepper rootstock tolerant to water stress i.e., NIBER^®^ (*Capsicum annuum* x C*. annuum*, abbreviated as N) was employed in this study. Two plant combinations were herein used: the commercial pepper variety “Maestral F1” (sweet pepper, California-type, Semillas Fitó, Spain, abbreviated as V) was grafted onto NIBER^®^ (abbreviated as V/N) and the self-grafted plants (abbreviated as V/V), thus considering the grafting effect. Early in March, the seeds of V and N were sown in 104 seedling trays filled with a peat-based substrate for germination. The grafts were performed after 2 months using the tube-grafting method (Penella et al., 2015). The grafted plants were maintained in a chamber with relative humidity above 95% and air temperature around 28-29°C for a 4-6 day period to successfully join rootstock and scion (Penella et al., 2014).

### 2.2 Hydroponic greenhouse conditions

Three weeks after grafting (by the beginning of June), seedlings were removed from the substrate and their roots were cleaned before being placed in 2L polyethylene pots in a Venlo-type greenhouse situated in Valencia (Spain, 39° 29’ 1.135” N 0° 20’ 27.315” W) under natural light conditions (610-870 µmol m^-2^ s^-1^) and temperature and relative humidity ranges of 21-25°C and 52-72%, respectively. Pots were filled with a nutrient solution containing (in mmol L^-1^): 13.0 NO_3_^-^, 1.0 H_2_PO_4_^-^, 2.45 SO_4_^2-^, 1.6 Cl^-^, 1.0 NH_4_^+^, 6.0 K^+^, 4.0 Ca^2+^, 2.5 Mg^2+^, 0.5 Na^+^ and micronutrients (15.8 µM Fe^2+^, 10.3 µM Mn^2+^, 4.2 µM Zn^2+^, 43.5 µM B^+^, 2.14 µM Cu^2+^), that were artificially aerated with an air pump. The electrical conductivity and pH of the nutrient solution were 2.1 dS m^-1^ and 6.7, respectively. After 7 days of seedling acclimation to the pots, the water stress treatment was initiated by adding 5% PEG 8000 (Sigma Co.) to the nutrient solution. The osmotic potential of the nutrient solutions, measured by a vapor osmometer (Digital osmometer, Wescor, Logan, USA), were -0.55 MPa for 5% PEG and -0.05 MPa for the control solution (0% PEG). The layout was a completely randomized design with 20 plants per combination (V/V and V/N) and treatment (5% PEG and control).

### 2.3 Fresh weight determination

Fresh weight determinations were performed at the end of the experiment (48h) using the plants that were not used for the hormonal analysis. Four plants per graft combination and treatment were analyzed by measuring the fresh weight of leaves and roots. The data are shown as a percentage of water stress over control conditions for self-grated (V/V) and variety grafted onto NIBER^®^ (V/N).

### 2.4 Photosynthesis analysis

Gas exchange measurements were performed at the beginning (T0) and the end of the experiment (T48). The gas exchange measurements were taken in the morning (9.30 am to 10.30 am GMT) with four plants per graft combination and treatment. The net CO_2_ assimilation rate (A_N_, μmol CO_2_ m^-2^ s^-1^), stomatal conductance (g_s_, mol H_2_O m^-2^ s^-1^), and transpiration rate (E, mmol H_2_O m^-2^ s^-1^) were determined on fully expanded leaves (3^rd^ - 4^th^ leaf from the apex) in the steady-state under saturating light conditions (1000 μmol m^-2^ s^-1^) and with 400 ppm CO_2_ by a LI-6400 infrared gas analyzer (LI-COR, Nebraska, USA) at 24 ± 2°C and 65 ± 10% relative humidity. Parameters A_N_/g_s_ and A_N_/E_leaf_ were calculated as intrinsic water use efficiency and instantaneous water use efficiency, respectively.

### 2.5 Sampling for hormonal analysis

The samples (leaves and roots) for hormonal analysis were taken before PEG addition (T0), and 4h (T4), 24h (T24), and 48h (T48) after water stress treatment began. Measurements were taken in fully expanded mature leaves (3^rd^ – 4^th^ leaf from the shoot apex) and 2 cm from distal roots. The layout was randomized with 4 samples of independent plants. The samples were frozen in liquid nitrogen immediately after harvest, conserved at −80°C, and afterwards freeze-dried.

### 2.6 Hormone extraction and analysis

Cytokinins (*trans*-zeatin, tZ, zeatin riboside, ZR, and isopentenyl adenine, iP), gibberellins (GA1, GA3, and GA4), indole-3-acetic acid (IAA), abscisic acid (ABA), salicylic acid (SA), jasmonic acid (JA) and the ethylene precursor 1-aminocyclopropane-1-carboxylic acid (ACC) were analyzed according to Albacete et al. (2008) and Großkinsky et al. (2014) with some modifications. Briefly, 40 mg of freeze-dried plant material were homogenized and dropped in 1 ml of cold (-20°C) extraction mixture of methanol/water (80/20, v/v). Solids were separated by centrifugation (20000 g, 15 min) and re-extracted for 30 min at 4°C in additional 1 mL of the same extraction solution. Pooled supernatants were passed through Sep-Pak Plus †C18 cartridge (SepPak Plus, Waters, USA) to remove interfering lipids and part of plant pigments and evaporated at 40°C under vacuum either to near dryness or until the organic solvent was removed. The residue was dissolved in 0.5 mL methanol/water (20/80, v/v) solution using an ultrasonic bath. The dissolved samples were filtered through 13 mm diameter Millex filters with 0.22 µm pore size nylon membrane (Millipore, Bedford, MA, USA).

Ten µL of filtrated extract were injected in a U-HPLC-MS system consisting of an Accela Series U-HPLC (ThermoFisher Scientific, Waltham, MA, USA) coupled to an Exactive mass spectrometer (ThermoFisher Scientific, Waltham, MA, USA) using a heated electrospray ionization (HESI) interface. Mass spectra were obtained using the Xcalibur software version 2.2 (ThermoFisher Scientific, Waltham, MA, USA). For quantification of the plant hormones, calibration curves were constructed for each analyzed component (1, 10, 50, and 100 µg L^-1^) and corrected for 10 µg L^-1^ deuterated internal standards. Recovery percentages ranged between 92 and 95%.

### 2.7 Statistical analysis

Data for each measure time (T0, T4, T24 and T48) and parameter were subject to an analysis of variance using Statgraphics Centurion 18 (Statgraphics Technologies, Inc., The Plains, Virgina, USA). The mean comparisons were performed using Fisher’s least significance difference (LSD) test at P ≤ 0.05.

## 3 Results

### 3.1 Fresh weight

**Figure 1:**
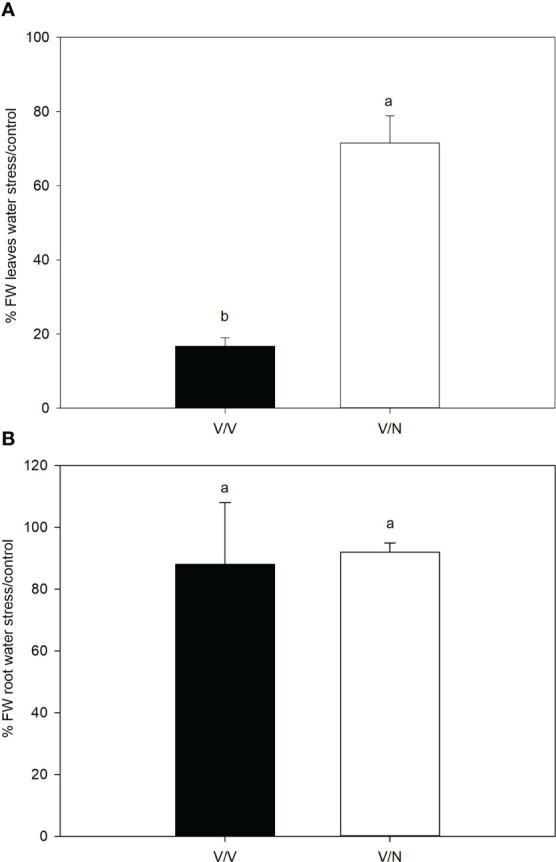
Percentage fresh weight in leaves **(A)** and roots **(B)** under water stress conditions compared to control conditions in the selfgrafted plants (V/V) and V grafted onto NIBER^®^ (V/N). Values are mean for n = 4. Different letters are statistically different according with LSD test (P ≤ 0.05).

The fresh weight of leaves (**Figure 1A**) was affected by water stress at the end of the experiment with significant differences for both plant combinations, with a 28% and 83% reduction in V/N and V/V respectively, compared with control conditions. The fresh root weight (**Figure 1B**) was less affected by water stress without significant differences, the reduction was 12% and 8% for V/V and V/N, respectively and respect to their controls.

### 3.2 Photosynthetic parameters

**Figure 2:**
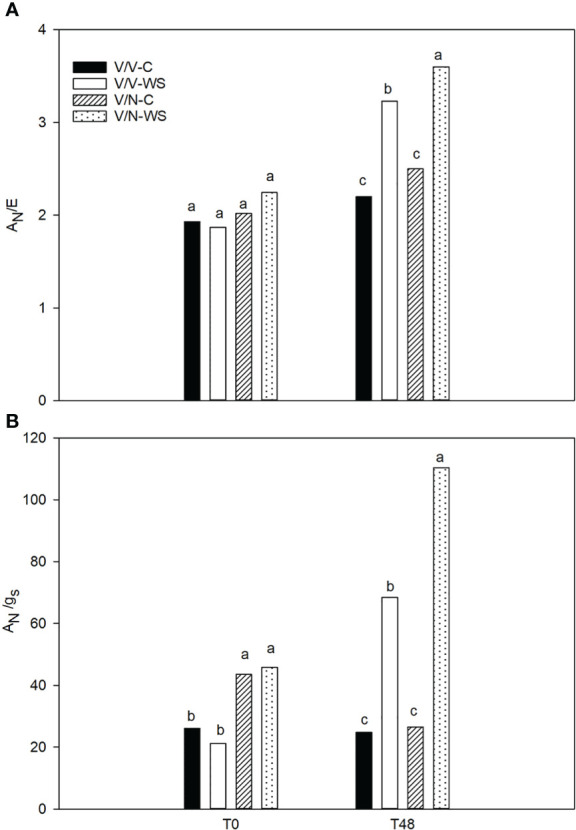
Instantaneous water use efficiency (A_N_/E) **(A)** and intrinsic water use efficiency (A_N_/g_s_) **(B)** in the self-grafted pepper plants (V/V) and the plants grafted onto NIBER^®^ (V/N) at 0% PEG (control, C) or 5% PEG (water stress, WS). Measurements were taken on T0 and T48 (hours after treatment with PEG began). Data are the mean values for n = 4. For each studied time, different letters indicate significant differences at P ≤ 0.05 (LSD test).

Instantaneous water use efficiency (A_N_/E) (**Figure 2A**) did not show significant differences at T0 for V/V and V/N with values between 1.8-2.2. After 48h, all plants with PEG treatment increased significantly the A_N_/E values. The increase with respect to its control plants was 47% for V/V and 44% for V/N, being the highest values for V/N-WS Regarding intrinsic water use efficiency (A_N_/g_s_) (**Figure 2B**), differences between genotypes were already observed at T0, V/V showed lower values compared with V/N. At the end of the experiment, plants under PEG treatment exhibited higher values with significant differences respect to control plants, plus the highest rise was observed in V/N plants.

### 3.3 Hormonal profiling

#### 3.3.1 ACC

**Figure 3:**
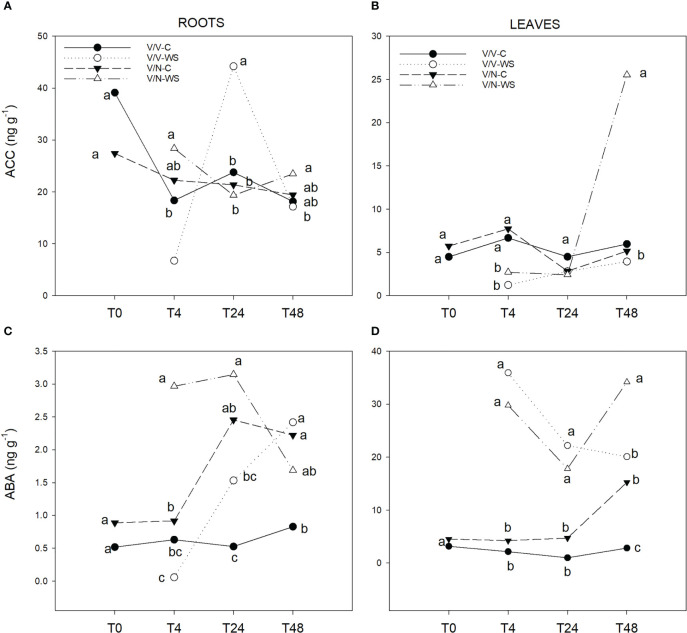
ACC **(A, B)** and ABA **(C, D)** levels in roots and leaves of self-grafted pepper plants (V/V) and the plants grafted onto NIBER^®^ (V/N) at 0% PEG (control,C) or 5% PEG (water stress, WS). Measurements were taken on T0, T4, T24 and T48 (hours after treatment with PEG began). Data are the mean values for n = 4. For each studied time, different letters indicate significant differences at P ≤ 0.05 (LSD test).

In general terms, ACC levels were higher in roots than in leaves, reaching up to 4.5-fold as a mean value for all times and all plant combinations. At T0, in the control treatment, V/V and V/N did not show significant differences either in roots or in leaves (**Figures 3A, B**). From T4 to T48, ACC concentration remained constant for each plant combination and treatment except at T24 for V/V in roots and at T48 for V/N in leaves, when the highest ACC levels were observed in response to water stress.

#### 3.3.2 ABA

In contrast to ACC, the ABA concentrations were higher in leaves than in roots. Similar to ACC at T0, ABA levels in roots and leaves (**Figures 3C, D**) did not display significant differences between V/V and V/N. In roots, at T4 and T24 the highest values were found in V/N-WS, while at the end of the experiment (T48) the ABA levels for this plant combination decreased by 54%. In leaves,V/N-WS ABA concentrations reached the highest values at T48 with significant differences. At T48 in roots and leaves, the lowest ABA values were found in V/V control plants, with significant differences to the rest of the plant combinations and treatments.

#### 3.3.3 IAA

**Figure 4:**
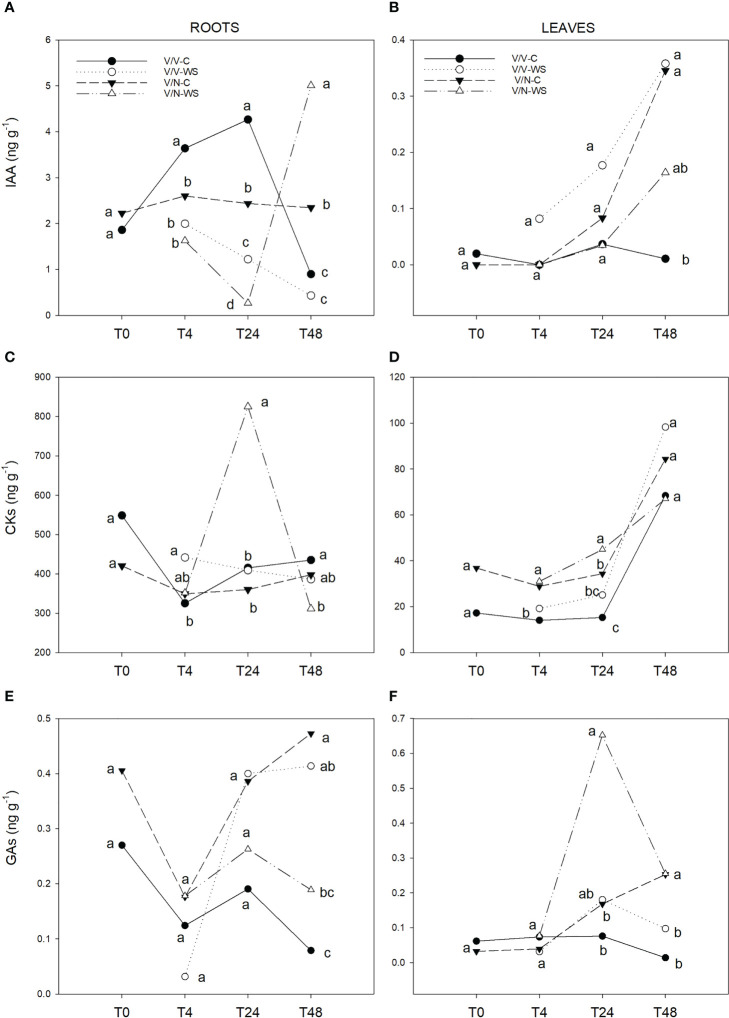
IAA **(A, B)**, CKs **(C, D)** and GAs **(E, F)** concentration in roots and leaves of self-grafted pepper plants (V/V) and the plants grafted onto NIBER^®^ (V/N) at 0% PEG (control, C) or 5% PEG (water stress, WS). Measurements were taken on T0, T4, T24 and T48 (hours after treatment with PEG began). Data are the mean values for n = 4. For each studied time, different letters indicate significant differences at P ≤ 0.05 (LSD test).

In roots, IAA concentration (**Figure 4A**) remained constant in V/N control and in V/V-WS decreased along the experiment. Nevertheless, for the rest of the plant combinations and treatments there was an erratic behavior, highlighting the IAA decrease from T24 to T48 for V/V control and the increase for V/N-WS. In leaves, IAA levels (**Figure 4B**) increased along the experiment (except for V/V control), reaching the maximum values at the end of the experiment in V/V-WS and V/N control, without significant differences with V/N-WS.

#### 3.3.4 CKs

CKs levels were 9-fold lower in leaves than roots, showing different dynamics in both organs. In roots, CKs levels (**Figure 4C**) remained constant after a decrease from T0 to T4, except for a sustained increase in V/N-WS at T24, following a decrease to the lowest CKs concentrations at the end of the experiment. In leaves (**Figure 4D**), CKs behavior resembled IAA role, with an increase from T4 to T48 for all plant combinations and treatments without significant differences between them at the end of experiment.

#### 3.3.5 GAs

The concentrations of GAs were similar in leaves and roots (**Figures 4E, F**). In roots, an increase was observed in V/N control and V/V-WS from T4 to T48. In leaves, in response to water stress, a peak of GAs was detected at T24 in V/N-WS, decreasing later to reach similar values to the control plants. The lowest GA values were found in V/V plants under control and water stress conditions.

#### 3.3.6 JA

**Figure 5:**
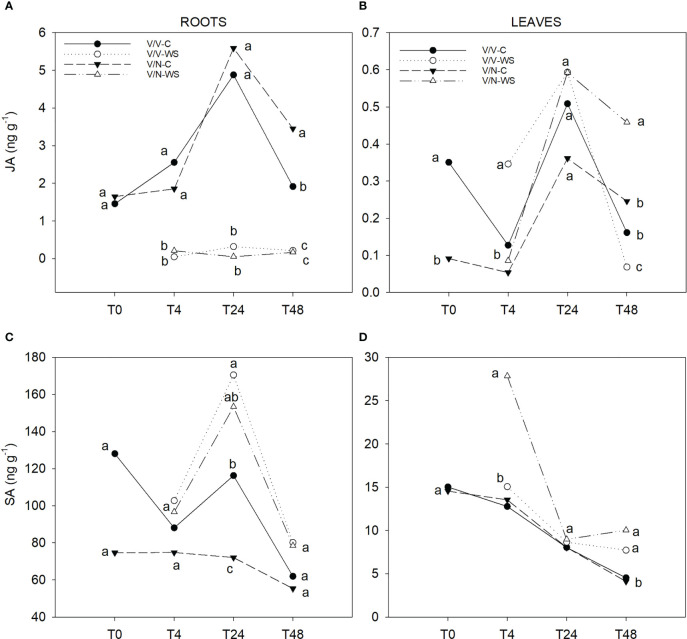
JA **(A, B)** and SA **(C, D)** levels in roots and leaves of self-grafted pepper plants (V/V) and the plants grafted onto NIBER^®^ (V/N) at 0% PEG (control, C) or 5% PEG (water stress, WS). Measurements were taken on T0, T4, T24 and T48 (hours after treatment with PEG began). Data are the mean values for n = 4. For each studied time, different letters indicate significant differences at P ≤ 0.05 (LSD test).

In roots, the levels of JA (**Figure 5A**) were 9-fold (as average) higher than in leaves. In roots, both treatments showed a differential trend. The highest values were observed in control conditions with a peak in both plant combinations at T24. Under water stress, V/V and V/N displayed the lowest values, without significant differences between them. However, in leaves (**Figure 5B**), a peak at T24 in the JA levels was observed for all plant combinations and treatments, following a decrease until T48, being the highest values for V/N-WS and the lowest values for V/V-WS, with significant differences.

#### 3.3.7 SA

In roots, increased SA concentrations in response to water stress were detected at T24 for V/V and V/N (**Figure 5C**). From T24 to T48 SA levels decreased to similar values for all plant combinations and treatments, without significant differences between them. A different evolution was observed in leaves (**Figure 5D**) with respect to roots, with a constant drop along the experiment in all plants and treatments, standing up V/N-WS with the highest concentration at T4. At the end of the experiment, two groups were separated, with highest SA levels belonging to water-stressed plants and lowest values to control plants.

## 4 Discussion

The study of hormone signaling fine-tuning during the early state of water stress exposure could help to distinguish and understand the tolerance responses in grafted plants mediated by efficient rootstocks. Indeed, plant hormones play a key role in controlling the adaptive processes to water stress, involving long-distance communication between different organs of plants together with *in situ* phytohormone synthesis (Albacete et al., 2008; Acosta-Motos et al., 2020; Lu et al., 2020).

Under water stress, roots are the first organs to perceive the osmotic stress, causing a rapid loss of shoot turgor and stomata closure, within minutes and hours (Munns, 2002). In this study, we observed at 48h that osmotic stress provoked a higher increase in A_N_/g_s_ in V/N than in V/V. This indicates that the regulation of stomatal closure is more efficient in the V/N combination, thus allowing major water accumulation in the leaves. In this sense, the fresh weight loss on leaves caused by osmotic stress was reduced in V/N plants compared to V/V, since N was considered as a tolerant rootstock. However, no effect was detected in the fresh weight of the roots in both graft combinations, indicating that the leaves were more sensitive to osmotic stress than the roots. Several authors (Hsiao and Xu, 2000; Sharp, 2002; López-Serrano et al., 2019) have also observed this differential response between roots and leaves. This is interesting because V/N water requirements and use should be lower, implicating positive economic and environmental effects. Indeed, graft technology has been used as an effective tool to increase WUE under water stress in vegetable crops like tomato (Cantero-Navarro et al., 2016; Gaion et al., 2018; Fullana-Pericàs et al., 2020), pepper (Gisbert-Mullor et al., 2020; Padilla et al., 2021) or cucumber (Liu et al., 2016).

Water relations traits are controlled by hormonal signals from root to shoot and shoot to root. Currently, ABA is the primary hormone that modulates stomatal performance contributing to the regulation of water-mediated stomatal closure and plays a key role in drought resistance (Holbrook et al., 2002; Gaion et al., 2018; Yang et al., 2022). Under short-term water stress, it has been described that ABA is synthesized mainly in the roots and afterward transported into guard cells to trigger stomata closure in leaves (Wilkinson and Davies, 2002; Allario et al., 2013; Sarwat and Tuteja, 2017). We found a fast induction (T4) of ABA concentration under water stress in V/N for roots and leaves, but only for the leaves in V/V. This suggests that the roots of a tolerant water stress rootstock such as NIBER^®^ were more sensitive to changes in the osmotic potential of the nutrient solution. As a consequence, V/N speeded up ABA synthesis in roots and transport to the leaves (Liu et al., 2016), as it can be observed in ABA rise at T48 in leaves coinciding with ABA decrease in roots. Hence, a differential rootstock behavior was observed under water stress, with an ABA hypersensitivity or biosynthesis to ABA in V/N plants that can increase WUE in the scion thus enhancing stress tolerance. Similar responses were observed in different vegetable-grafted plants using tolerant rootstocks under water stress, such as cucumber grafted onto luffa (Liu et al., 2016) and tomato grafted onto different recombinant inbred lines from *Solanum pimpinellifolium* (Cantero-Navarro et al., 2016). Considering that the ABA levels in leaves were higher than in the roots in all plant combinations and treatments, the synthesis of ABA in the scion cannot be ruled out (Manzi et al., 2017; López-Serrano et al., 2020).

ABA and ethylene (or its precursor ACC) regulate stress responses in coordinated ways, in senescence, flooding, drought, and wounding stresses, and have been considered important WUE regulators under stress conditions (Wilkinson, 2004; Cantero-Navarro et al., 2016). However, the interaction between ethylene and ABA in relation to stomata closure is controversial and still not fully understood (Wilkinson et al., 2012; Chen et al., 2013). Generally, ABA and JA are positive regulators of stomata closure, while IAA and CKs have been described as negative regulators. However, the regulatory role of ethylene on stomata behavior is ambiguous, acting as a positive or negative regulator depending on the tissue and environmental conditions (Nemhauser et al., 2006; Huang et al., 2008; Daszkowska-Golec and Szarejko, 2013). In this sense, under water stress, elevated ABA levels usually limited ethylene production in maize plants (Sharp et al., 1994). In *Arabidopsis thaliana*, ethylene physiologically inhibited ABA-dependent stomata closure through the ethylene signaling pathway (Tanaka et al., 2005). Despite the apparent antagonist relation between ABA and ethylene under water stress (Spollen et al., 2000), in *A. thaliana* ethylene signaling was promoted during short-term ABA treatment (*ERF1*, *EDF1* and *EDF4* up-regulated) (Yang et al., 2014). In citrus (Tudela and Primo-Millo, 1992) and pea (Belimov et al., 2009), water stress induced an increase in ACC concentrations. Additionally, in grafted tomato plants, ACC in the roots could increase agronomic WUE (Cantero-Navarro et al., 2016). These results show that ethylene also plays an important role in stomatal control (Desikan et al., 2006; Vysotskaya et al., 2011). We did not find dramatic changes in ACC levels in leaves, except for an important increase at the end of the experiment (after 48h of water stress) in plants grafted onto NIBER^®^, coinciding with a significant rise of intrinsic WUE and ABA. These results could indicate that ACC is promoted at the initial stage of ABA-dependent control of water stress, using ACC as a rapid response to accelerate tolerance in V/N (Yang et al., 2014). Importantly, this effect was not observed in V/V plants, and only a maximum ACC concentration was measured at T24 in roots following an important decrease at T48h. This drop was not associated with an increase in leaves, which could indicate ACC degradation in the roots.

In addition to ABA and ethylene, JA and SA are also involved in the stomata response under water stress (Nazareno and Hernandez, 2017; Munemasa et al., 2019; Müller and Munné-Bosch, 2021). JA and 12-OPDA (JA precursor) are positive regulators of stomata closure, leading to increased drought stress tolerance (Savchenko and Dehesh, 2014). However, we did not find any change in JA concentrations in the root system under water stress, indicating that JA is not a primary hormonal factor controlling drought stress and/or there was an early transient increase. Similarly, other studies did not find changes in JA under water stress, possibly due to JA accumulation being characterized as early transient (within 3h), therefore being dependent on the measure time (Luo et al., 2019; Wang et al., 2020; Huntenburg et al., 2022). In the leaves, the highest JA levels at the end of the experiment were found in V/N under water stress, which coincides with increased levels of ABA and ACC and stomata closure. Regarding SA, its role has been associated with biotic stress defense responses (Vlot et al., 2009). Recently, different research works have suggested that SA can have an important contribution to abiotic stress-induced signaling and tolerance (Miura and Tada, 2014; Zandalinas et al., 2016). However, the effect of SA on water stress tolerance is still unclear (Borsani et al., 2001). In our experimental conditions, SA increased at T24h in the roots mainly under water stress in both plant combinations, thus indicating that SA may be involved in drought responses. SA content augmented approximately 2-fold with water stress in barley roots associated to ABA increase (Bandurska and Stroinski, 2005), corresponding to our observations at T24h. In leaves, SA has been described to be implicated in stomata closure (Mori et al., 2001; Liu et al., 2013; Prodhan et al., 2018), and in the enhancement of antioxidants and antioxidant enzymes mainly to protect the photosynthetic apparatus (Miura and Tada, 2014; Khan et al., 2015; Zandalinas et al., 2016). The endogenous SA accumulation in leaves has been detected in several crops like citrus (Zandalinas et al., 2016), mustard (Alam et al., 2013), and *Phyllyrea angustifolia*, where SA levels were correlated with the water stress degree, increasing up to 5-fold under severe stress, thus suggesting a role for SA in drought tolerance (Munné-Bosch and Peñuelas, 2003). In pepper leaves, a drastic SA increase occurred immediately after water stress was applied only in V/N plants. Afterwards, SA concentrations decreased to reach values similar to V/V values, but higher than V/N control plants. This could indicate that SA accumulation is related to water stress, but it is also dependent on the rootstock genotype.

IAA, CKs, and GAs are hormones related to plant growth and development, and they are also involved in regulating drought responses (Devireddy et al., 2021; Raza et al., 2022). However, the variations of these hormones content under water stress are contradictory in our experiment. In roots under water stress, IAA content showed a gradual decline in V/V from T4 until the end of the experiment, but in V/N plants the IAA decrease occurred at T24 and, thereafter, IAA concentration increased up to a maximum. Regarding the concentrations of CKs under water stress, a transient increase at T24 in V/N was observed in roots and then CK levels declined to reach values similar to the optimal watering conditions and to the rest of the plant combinations and treatments. In both hormones, the highest concentrations were linked to water stress and to water stress tolerant rootstock (NIBER^®^), but this effect was not observed for GAs. IAA and CKs promote root branching and root growth, having a potential role in drought-tolerance mechanisms (Verma et al., 2016; Ullah et al., 2018). By using NIBER^®^ as rootstock under salinity conditions for 10 days, a significant increase in root length was stated (López-Serrano et al., 2020), which could explain the increase in IAA and CKs when NIBER^®^ is used under the osmotic treatment. In addition, increasing endogenous IAA levels in roots under osmotic stress have been associated with enhanced tolerance in Arabidopsis (Kim et al., 2013) and *Prosopis strombulifera* (halophyte) (Llanes et al., 2014) due to an increase in lateral root formation and enlarged root system (Llanes et al., 2016).

However, the GA trend in roots did not seem to be dependent on either water stress or rootstock genotype, considering that there were no significant differences between V/V and V/N at the end of the experiment.

In the leaves, IAA and CK levels increased along the experiment, but no significant differences were observed between both rootstock and treatments, which could indicate a poor relation with water stress. Increasing IAA in maize leaves was observed on the first day under water stress (provoked by PEG addition, -0.4MPa) (Wang et al., 2008) with an osmotic potential similar to the one used in this experiment. The increase of CKs has been related to an amelioration of the effect of water stress by stimulating osmotic adjustment and allowing water absorption. However, the increase in IAA and CKs in the majority of studies is associated with stimulated stomata opening and they are considered as ABA antagonists (Pospíšilová, 2003; Gaion et al., 2018). The stomata closure observed in our study could be the consequence of crosstalk between concentration and action place (Ullah et al., 2018; Iqbal et al., 2022).

Regarding GAs in leaves, they can modulate drought responses through stomata development and responses (Nir et al., 2014; Gaion et al., 2018). In our results, an important transient increase in leaves at T24 was recorded in V/N under water stress. Subsequently, GA levels declined to reach control values, and no differences in GA content associated with water stress were observed at the end of the experiment, although there were significant differences between rootstocks. Several studies have demonstrated that the reduction of GA levels contributes to plant growth restriction under drought (Llanes et al., 2016). Besides, in halophyte and some no-halophyte tolerant plants, GA concentrations in leaves increased in response to an osmotic potential decrease to maintain the growth (Li et al., 2012; Colebrook et al., 2014; Llanes et al., 2016). The transient increase observed in V/N under water stress could be associated with GA modulation and signaling for growth preservation.

The knowledge about endogenous phytohormone modulation in response to water stress remains scarce given that most plants’ hormonal studies are based on exogenous applications. Overall, this work reflects the fast modulation of the balance of major phytohormones during short-term water stress in young pepper plants, self-grafted or grafted onto a water stress tolerant rootstock such as NIBER^®^. Phytohormone levels during early water stress exposure (up to 48h) revealed natural variability present in V/V and V/N and how V/N integrates various hormonal signals to tolerate drought imposition. It is essential to determine the water stress tolerance mechanisms and to find the key factors responsible for short-term tolerance, such as hormones. Therefore, this study will allow to understand the early differential responses to water stress in grafted pepper plants and the contribution of NIBER^®^ rootstock hormonal balance to scion water stress improvement. This study will be crucial to extend knowledge and open the door to future biotechnological strategies to improve drought tolerance. However, due to the high level of complexity of the phytohormones network, further studies are required.

## Data availability statement

The original contributions presented in the study are included in the article/supplementary material. Further inquiries can be directed to the corresponding author. The authors declare that all data generated during this work are included in the manuscript.

## Author contributions

YP: conducted experiments, methodology, investigation, data curation, review. RG-M: conducted experiments, investigation, review. SL-G: Conceptualization, investigation, review. PM-M: methodology, investigation, analytical tools, ÁC and AA: Conceptualization, methodology, investigation, writing – review & editing. All authors contributed to the article and approved the submitted version.
